# The association between genetic variants of *angiopoietin-like 3* and risk of diabetes mellitus is modified by dietary factors in Koreans

**DOI:** 10.1038/s41598-018-36581-z

**Published:** 2019-01-25

**Authors:** Clara Yongjoo Park, Jiyoung Moon, Garam Jo, Juhee Lee, Oh Yoen Kim, Hannah Oh, Hyunjung Lim, Min-Jeong Shin

**Affiliations:** 1 Department of Food and Nutrition, Human Ecology Research Institute, Chonnam National University, Gwangju, 61186 Republic of Korea; 2 Department of Public Health Sciences, BK21PLUS Program in Embodiment: Health-Society Interaction, Graduate School, Korea University, Seoul, 02841 Republic of Korea; 3 Department of Food Science and Nutrition, Dong-A University, Busan, 49315 Republic of Korea; 4 Department of Medical Nutrition, Graduate School of East-West Medical Science, Kyung Hee University, Yongin, 17104 Republic of Korea

## Abstract

The role of angiopoietin-like 3 (ANGPTL3) in blood lipid levels, cardiovascular disease risk, and glucose metabolism has received wide attention. This study aimed to examine whether rs11207997 in *ANGPTL3* is associated with a 10-year risk of diabetes mellitus (DM) and if the association is modified by the consumption of certain food groups or nutrients. A prospective cohort study was designed using the Ansan–Ansung data of the Korean Genome and Epidemiology Study (*n* = 7,358; age ≥40 years at baseline). Participants with the T allele of rs11207997, particularly TT homozygotes, had lower triglyceride (TG) and total cholesterol levels than those with CC. There was no association with fasting blood glucose or other biochemical parameters. *ANGPTL3* mRNA was positively associated with circulating TG levels and blood pressure (all *p* < 0.05). Cox proportional hazard models showed that the rs11207997 T allele is associated with a lower risk of DM after adjusting for covariates (hazard ratio: 0.90, 95% confidence interval: 0.812–0.998, *p* = 0.046). Furthermore, the association between rs11207997 and the risk of DM was modified by dietary factors. These associations were no longer statistically significant when additionally adjusted for baseline TG, a potential mediator. Our data suggest that genetic variation of rs11207997 in the *ANGPTL3* gene is associated with risk of DM, possibly through contributing to a lifelong set point of TG.

## Introduction

Angiopoietin-like proteins (ANGPTLs) represents a family of proteins that are involved in various physiological and pathological processes^1^ including lipid and glucose metabolism^2^, inflammation^3^, hematopoietic stem cell activity^4^, and cancer cell invasion^5^. Among the eight ANGPTLs discovered to date^1^, ANGPTL3 plays an important role in the regulation of plasma triglyceride^6^ and cholesterol levels, mainly via reversible inhibition of lipoprotein lipase (LPL) activity^6–8^. Recently, ANGPTL3 has been considered an emerging target of novel drug therapy for cardiovascular disease (CVD)^6–8^. Deficiency of ANGPTL3 is associated with hypolipidemia and reduced risk of CVD in Caucasians^9–11^. Although the precise mechanism is not yet fully understood, ANGPTL3 is thought to inhibit the activity of LPL and endothelial lipase, thereby increasing blood lipid levels^12^. ANGPTL3 levels are also associated with insulin sensitivity and glucose metabolism^12,13^. High levels of hepatic ANGPLT3 mRNA and protein have been observed in insulin-resistant and insulin-deficient mice^13^. Reportedly, loss-of-function mutations of *ANGPTL3* in Italians result in lower levels of plasma insulin and glucose as well as homeostatic model assessment of insulin resistance (HOMA-IR)^12^.

However, the results of studies examining the involvement of ANGPTL3 in metabolic regulation are inconsistent, possibly due to differences in age and race of the study participants. For example, it has been shown that circulating ANGPTL3 levels are associated with fasting insulin levels and HOMA-IR but not with triglyceride (TG) or cholesterol levels in Korean boys and girls^14^, whereas the association has not been investigated in Korean adults. In Chinese boys and girls, rs1748195 polymorphisms in the *ANGPTL3* gene are not associated with blood lipid levels, including TG, total cholesterol (TC), high-density lipoprotein cholesterol (HDLC), or low-density lipoprotein cholesterol (LDLC)^15^. In contrast, among the participants of the Healthy Lifestyle in Europe by Nutrition in Adolescence study, both adolescents and adults with the T minor allele of rs11207997 in *ANGPTL3* had lower HDLC and apolipoprotein A-1 levels than those carrying two C alleles^16^. However, no further investigations regarding the influence of the genetic variation of rs11207997 in *ANGPTL3* on glucose metabolism and clinical endpoints including diabetes mellitus (DM) risk have been reported.

Based on the reported associations between ANGPLT3 and metabolic profiles, we hypothesized that rs11207997 in *ANGPTL3* is associated with life-long metabolic exposure to DM. In this study, using data from the Ansan–Ansung (urban–rural) cohort with a median follow-up of 10 years, we prospectively examined the associations of rs11207997 with DM risk among 7,358 Korean older adults. We also investigated whether the association is modified by the consumption of certain food groups previously reported to significantly affect metabolic status and the risk of DM.

## Results

### Characteristics of study participants

After a median follow-up of 9.8 years, 1,168 participants (15.9%) were diagnosed with DM (Table 1). Participants who developed DM within 10 years were more likely to be smokers, possibly due to the higher proportion of male individuals, were older, and had a higher mean body mass index (BMI), systolic blood pressure (SBP), diastolic blood pressure (DBP), TG, TC, LDLC, fasting blood glucose (FBG), hemoglobin A1c (HbA1c), HOMA-IR and lower HDLC at baseline than non-DM participants. The genotype distribution for rs11207997 (*ANGPTL3*) polymorphisms was as per the Hardy–Weinberg equilibrium. The minor allele (T) frequency was approximately 0.221 in all participants. The genetic variants of rs11207997 in *ANGPTL3* were significantly associated with circulating TG and TC levels (Table 2). Participants with the T minor allele had lower TG and TC levels (CC: 154.6 ± 1.2, CT: 147.8 ± 1.5, TT: 140.5 ± 3.5 mg/dL for TG, *p* < 0.001; CC: 190.2 ± 0.5, CT: 189.7 ± 0.7, TT: 185.5 ± 1.7 mg/dL for TC, *p* = 0.029).

**Table 1:** Baseline characteristics of the study population.

	Total (*n* = 7,358)	10-year follow-up		
		Healthy (*n* = 6,190)	Diabetes mellitus (*n* = 1,168)	*p-* value
Age, years	51.6 ± 0.1	51.3 ± 0.1	53.1 ± 0.3	<0.001
Male, % (n)	46.6 (3,427)	45.4 (2,812)	52.7 (615)	<0.001
Area, % (n)				0.001
Ansung (rural)	46.9 (3,452)	47.7 (2,954)	42.67 (498)	
Ansan (urban)	53.1 (3,906)	52.3 (3,236)	57.4 (670)	
Body mass index, kg/m^2^	24.45 ± 0.04	24.29 ± 0.04	25.30 ± 0.09	<0.001
Total energy intake, kcal	1957.6 ± 8.4	1962.2 ± 9.3	1933.5 ± 20.0	0.216
Income level, % (n)				0.993
Lowest	33.9 (2,450)	33.8 (2,057)	34.2 (393)	
Lower-middle	29.4 (2,124)	29.4 (1,787)	29.3 (337)	
Upper-middle	29.1 (2,103)	29.2 (1,772)	28.8 (331)	
Highest	7.6 (548)	7.6 (460)	7.7 (88)	
Education level, % (n)				0.389
≤Elementary school	31.8 (2,316)	31.4 (1,929)	33.4 (387)	
≤Middle school	23.2 (1,692)	23.4 (1,438)	21.9 (254)	
≤High school	31.6 (2,305)	31.8 (1,951)	30.6 (354)	
≥University	13.4 (981)	13.3 (818)	14.1 (163)	
Current smoking, % (n)	25.5 (1,848)	25.0 (1,524)	28.1 (324)	0.025
Current drinking, % (n)	47.7 (3,474)	47.1 (2,887)	50.7 (587)	0.024
Metabolic equivalent, hours/day	19.4 ± 0.2	19.4 ± 0.2	19.1 ± 0.5	0.531
Biochemical markers				
SBP, mmHg	120.4 ± 0.2	119.5 ± 0.2	125.2 ± 0.5	<0.001
DBP, mmHg	79.8 ± 0.1	79.3 ± 0.1	82.5 ± 0.3	<0.001
TG, mg/dL^§^	151.5 ± 0.9	145.3 ± 0.9	184.8 ± 2.7	<0.001
TC, mg/dL	189.8 ± 0.4	188.4 ± 0.4	197.3 ± 1.0	<0.001
HDLC, mg/dL	45.0 ± 0.1	45.4 ± 0.1	43.0 ± 0.3	<0.001
LDLC, mg/dL	115.0 ± 0.4	114.5 ± 0.4	118.3 ± 1.0	<0.001
FBG, mg/dL	83.0 ± 0.1	82.0 ± 0.1	88.2 ± 0.3	<0.001
HbA1c, %	5.571 ± 0.005	5.518 ± 0.004	5.855 ± 0.014	<0.001
HOMA-IR	1.54 ± 0.01	1.51 ± 0.01	1.73 ± 0.03	<0.001
Genotype distribution				
rs11207997				
CC/CT/TT, %	60.8/34.3/4.9	60.3/34.7/5.0	63.4/32.1/4.5	0.149
Minor allele frequency	0.221	0.223	0.206	

Data are given as mean ± standard error or as percentages for continuous and categorical variables. Differences in the genotype were determined using Student’s t-test and chi-square tests. ^§^Tested after log-transformation. DBP, diastolic blood pressure; FBG, fasting blood glucose; HDLC, high-density lipoprotein cholesterol; HOMA-IR, homeostatic model assessment of insulin resistance; LDLC, low-density lipoprotein cholesterol; SBP, systolic blood pressure; TC, total cholesterol; TG, triglyceride.

**Table 2:** Clinical parameters of the population according to rs11207997 (*ANGPTL3*) genotype at baseline.

rs11207997	C/C (*n* = 4,474)	C/T (*n* = 2,522)	T/T (*n* = 362)	*p*-value
SBP, mmHg (*n* = 7,358)	120.5 ± 0.3	120.3 ± 0.4	121.1 ± 1.0	0.968
DBP, mmHg (*n* = 7,358)	79.8 ± 0.2	79.6 ± 0.2	80.3 ± 0.6	0.970
TG, mg/dL^§^ (*n* = 7,357)	154.6 ± 1.2^a^	147.8 ± 1.5^b^	140.5 ± 3.5^b^	<0.001
TC, mg/dL (*n* = 7,357)	190.2 ± 0.5^a^	189.7 ± 0.7^ab^	185.5 ± 1.7^b^	0.029
HDLC, mg/dL (*n* = 7,357)	45.1 ± 0.2	45.0 ± 0.2	44. 8 ± 0.5	0.561
LDLC, mg/dL (*n* = 7,240)	114.8 ± 0.5	115.7 ± 0.6	113.0 ± 1.5	0.956
FBG, mg/dL (*n* = 7,329)	83.1 ± 0.1	82.9 ± 0.2	83.0 ± 0.5	0.749
HbA1c, % (*n* = 7,356)	5.58 ± 0.01	5.56 ± 0.01	5.57 ± 0.02	0.676
HOMA-IR (*n* = 7,329)	1.55 ± 0.01	1.54 ± 0.02	1.51 ± 0.04	0.769

Data are presented as mean ± standard error for clinical parameters. Differences among genotypes were determined by analysis of variance. Significance was tested using the generalized linear model with Bonferroni’s multiple comparisons after adjusting for age, sex, location, total energy intake, body mass index, physical activity, education level, and drinking status. ^§^Tested after log-transformation. DBP, diastolic blood pressure; FBG, fasting blood glucose; HDLC, high-density lipoprotein cholesterol; HOMA-IR, homeostatic model assessment of insulin resistance; LDLC, low-density lipoprotein cholesterol; SBP, systolic blood pressure; TC, total cholesterol; TG, triglyceride.

### *ANGPTL3* mRNA expression in LCLs is associated with circulating TG levels and BP

**Figure 1:**
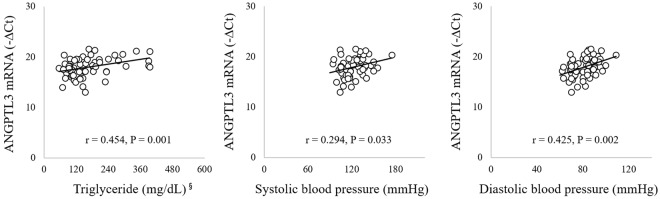
Relationship between *ANGPTL3* mRNA expression (-ΔCt) and circulating triglyceride levels (mg/dL) or blood pressure (mmHg). r: correlation co-efficient; P: p-value. Data were tested by partial correlation analyses adjusted for age, sex, location, body mass index, total energy intake, physical activity, education level, and drinking status ^§^log-transformed for the analysis.

A linear regression analysis was performed in a subset of 62 healthy participants (24 males and 38 females, mean age: 54.5 ± 1.2 years) to determine if *ANGPTL3* mRNA expression is associated with metabolic parameters and BP. Upregulated *ANGPTL3* expression in lymphoblastoid cell lines (LCLs) was positively correlated with circulating TG levels (r = 0.454, *p* = 0.001), SBP (r = 0.294, *p* = 0.033), and DBP (r = 0.425, *p* = 0.002) after adjusting for age, sex, location, BMI, total energy intake, physical activity, education level, and drinking status (Fig. 1). However, *ANGPTL3* mRNA expression was not correlated with glycemic parameters (i.e., FBG, HOMA-IR, and HbA1c) or other circulating lipid levels (i.e., TC, LDLC, and HDLC) (data not shown).

### Variants of rs11207997 are associated with the risk of DM

To investigate the effect of rs11207997 genotype on the risk of developing DM, Cox proportional regression was performed with adjustments for potential covariates (i.e., age, sex, location, total energy intake, physical activity, education level, and smoking and drinking status). The presence of the T minor allele was associated with a 10% lower risk of DM (hazard ratio (HR) = 0.901, 95% confidence interval (CI) = 0.812–0.998, *p* = 0.046; Table 3). When we evaluated the contribution of circulating TG levels, a potential intermediate variable, in the association between rs11207997 and DM, by additionally adjusting for them in the models, the association was attenuated and no longer statistically significant (HR = 0.936, 95% CI = 0.845–1.036, *p* = 0.200).

**Table 3:** Association between rs11207997 (*ANGPTL3*) polymorphism and diabetes occurrence.

rs11207997	Diabetes	*p*-value
Case	1,168	
Total person-years	43660.40	
HR (95% CI)	0.901 (0.812–0.998)	0.046

Statistical differences were obtained by Cox regression analysis adjusted for age, sex, location, total energy intake, body mass index, physical activity, education level, smoking and drinking status. CI, confidence interval; HR, hazard ratio.

### Association between rs11207997 genotype and the risk of DM is modulated by food consumption patterns

Table 4 presents the association between rs11207997 genotype and the risk of DM according to food group consumption quartiles (Q1–Q4). The median values of fruit intake quartiles were 3.6, 9.6, 16.8, and 28.6 servings/week, respectively. Among the participants in the Q2 group, T minor allele carriers had a lower HR for DM than CC homozygotes (HR: 0.795, *p* = 0.032). Regarding vegetable intake, the mean intake of participants in the Q2 group (39.2 servings/week; range: 32.6–45.3 servings/week) was slightly lower than that recommended for Koreans (≥6 servings/d^17^ or ≥42 servings/week). Among participants in this group, HR for DM was lower when carrying the T allele (HR: 0.793, *p* = 0.030). However, an association between genotype and the risk of DM was not observed when intake was above or below Q2 for vegetables. Regarding unprocessed meat consumption, T minor allele carriers in the Q4 group had a lower HR for DM than C major allele homozygotes (HR: 0.808, *p* = 0.042). Unprocessed meat consumption in the study participants was generally low, and the Q4 group consumed approximately 1 serving/d. As for sodium intake, the mean intake in the Q1 group (1600 mg/d) was greater than the recommended intake for Koreans (1500 mg/d)^17^. The maximum intake of participants in the Q1 group exceeded the “goal” for sodium intake (≤2000 mg/d^17^). However, the T allele in the Q2 group, but not in other quartiles, was associated with a lower HR for DM (mean: 2500 mg/d, range: 2100–2830 mg/d, HR: 0.804, *p* = 0.036). Among the participants consuming 0.06–0.99 servings/week of milk (Q2), the T minor allele carriers had a significantly lower HR for DM (HR: 0.800, *p* = 0.300).

**Table 4:** Stratification analysis by dietary factors for associations of rs11207997 genotype (*ANGPTL3*) with diabetes occurrence.

	Distribution for dietary risk factors					HR^†^	95% CIs
	*N*	Mean	Median	Min	Max		
Fruits (servings/week)							
Q 1	1,839	3.5	3.6	0	6.51	0.828	0.656–1.038
Q 2	1,838	9.6	9.6	6.52	13.00	0.795	0.644–0.980
Q 3	1,841	16.9	16.8	13.00	21.42	1.139	0.942–1.377
Q 4	1,840	32.8	28.6	21.43	147.00	0.825	0.676–1.005
Vegetables (servings/week)							
Q 1	1,839	22.8	24.8	0	32.57	0.823	0.663–1.021
Q 2	1,840	39.2	39.3	32.57	45.32	0.793	0.643–0.978
Q 3	1,839	52.2	51.8	45.34	60.43	1.050	0.867–1.272
Q 4	1,840	82.6	74.3	60.44	310.65	0.927	0.758–1.134
Whole grains (servings/week)							
Q 1	1,834	0.1	0.0	0	0.29	0.920	0.742–1.141
Q 2	1,830	3.0	2.0	0.29	7.38	0.916	0.738–1.137
Q 3	1,796	10.7	10.6	7.40	13.94	0.865	0.714–1.048
Q 4	1,898	20.2	21.0	14.00	43.50	0.888	0.729–1.082
Unprocessed meat (servings/week)							
Q 1	1,829	0.6	0.7	0	1.26	0.946	0.774–1.155
Q 2	1,847	1.9	1.9	1.27	2.63	0.868	0.707–1.066
Q 3	1,842	3.5	3.5	2.63	4.71	0.945	0.769–1.161
Q 4	1,840	8.5	6.8	4.71	103.91	0.808	0.658–0.992
Sodium (g/day)							
Q 1	1,821	1.6	1.6	0.14	2.10	0.849	0.685–1.052
Q 2	1,822	2.5	2.5	2.10	2.83	0.804	0.656–0.986
Q 3	1,822	3.2	3.2	2.83	3.67	0.976	0.803–1.186
Q 4	1,822	4.9	4.4	3.67	15.31	0.965	0.786–1.184
Milk (servings/week)							
Q 1	2,053	0	0	0	0	0.907	0.741–1.111
Q 2	1,858	0.5	0.4	0.06	0.99	0.800	0.654–0.978
Q 3	2,020	2.5	2.5	1.02	3.50	1.029	0.845–1.251
Q 4	1,427	6.4	5.3	3.56	31.50	0.865	0.693–1.080

Statistical differences were obtained by Cox regression analysis. ^†^Adjusted for age, sex, location, total energy intake, body mass index, physical activity, education level, and drinking status. CI, confidence interval; HR, hazard ratio; Q, quartile.

## Discussion

The present study demonstrates that the minor allele of rs11207997 in *ANGPTL3* is associated with a lower incidence of DM, and the genetic effect on the risk of DM can be modified by dietary factors, such as the intake of fruits, vegetables, unprocessed meat, sodium, and milk, in older Korean adults. Compared with CC homozygotes, participants with the T allele of rs11207997 had lower TG and TC levels at baseline, and lower risk of DM. This genotype effect on DM risk is possibly mediated through chronically reduced levels of circulating TG. The inverse association between T minor allele of rs11207997 and DM was restricted to those with Q2 intakes of fruits, vegetables, milk, or sodium and those with relatively high (Q4) intakes of unprocessed meat.

The specific role of rs11207997 in ANGPTL3 function and the risk of DM has not yet been elucidated. In our participants, the protective effect of the T minor allele of rs11207997 on DM-related biochemical markers (i.e., FBG, HbA1c, and HOMA-IR) was not present at baseline, but T minor allele carriers had a lower risk of DM in our 10-year follow-up. The function of rs11207997 on glucose metabolism may be partly explained by the report by Wang *et al*.^18^. Mice lacking ANGPTL3 had a lower uptake of VLDL-TG, but fat mass was preserved by a 10-fold increase in glucose uptake and *de novo* synthesis of white adipose tissue^18^. That is, the genetic variants of rs11207997 in *ANGPTL3*, which regulates lipid metabolism (TG and TC), may consecutively affect glucose uptake and fat accumulation in adipose tissue, resulting in future alteration in glycemic status, insulin sensitivity, and DM development. In our participants, circulating TG and TC levels at baseline were lower in participants carrying the T allele and TG was positively associated with *ANGPTL3* mRNA levels, regardless of genotype, and the genotypic associations were attenuated when additionally adjusted for TG. Further research is required to clarify the underlying mechanism of the genetic variants of rs11207997 on DM-related metabolic status.

In our Korean older adult population, the effect of the genetic variants of rs11207997 on the risk of DM was also modulated by the amounts of intakes of certain types of food. Diet is associated with CVD risk, possibly through its effect on lipid and glucose metabolic profiles, systemic inflammation^19^, and DM^20^. We observed lower HRs for DM in T minor allele carriers of rs11207997 at or near the recommended intakes for fruits, vegetables, and sodium. Regarding fruit and vegetable intakes, the effect of genetic variants on the risk of DM was observed in participants in the Q2 group, which encompasses the recommended intakes for Koreans, whereas the risk of DM in those with intakes above or below the recommendations was not associated with genotype. An association between genotype and the risk of DM was evident in participants with sodium intakes slightly above the recommendations of the World Health Organization and Dietary Reference Intakes for Koreans (<2000 mg/d), which is well below the mean sodium intake of Koreans (~4800 mg/d^21^). Caution is required in the interpretation of data regarding sodium intake. Analyzed intake data may not accurately reflect the participants’ actual intake because the Standard Tables of Food Composition of Korea^22^ was utilized to assess sodium intake, whereas sodium use may vary greatly depending on the habits of those who prepare and cook food. Thus, food intake may affect the genetic risk of DM, but the results of this study may not apply to populations with lower sodium intake.

No specific dietary recommendations for Koreans on milk alone or unprocessed meat are available. Milk intake in our population was very low (mean intake: 1.0 serving/week), compared with the Korean recommendation of 1–2 servings of dairy products/day. Calcium intake may not be the critical factor for the interaction effect between milk intake and genotype on the risk of DM in the present study. Milk is a good source of calcium and has been reported to affect metabolic status in association with certain single nucleotide polymorphisms (SNPs)^23^. However, approximately 14 mg of Ca/d is provided from milk when intake is within Q2 (mean intake: 0.5 servings/week), from which a significant association between rs11207997 genotype and risk of DM was observed. It seems more likely that the interaction effect between milk intake and genotype on the risk of DM is due to other factors in milk (i.e., lactose, protein, fat, phosphorus, potassium, and other nutrients) rather than calcium alone^24^. In contrast, we found that the T allele of rs11207997 decreases the HR for DM when unprocessed meat consumption is relatively high (Q4). Beef, pork, chicken, and processed meat consumption were not associated with metabolic syndrome risk in Koreans using Korea National Health and Nutrition Examination Survey (KNHANES) data^25^, but the associations may have been masked by genotype. Meat is a good source of cholesterol and saturated fat, in addition to protein, vitamins, and minerals. Although no dietary guidelines are provided for unprocessed meat alone, the mean unprocessed meat intake in the Q4 group was approximately one quarter of the current recommendations for meat, fish, eggs, and legume intakes for Koreans (4 servings/day^17^). Therefore, the results of the present study may not agree with results in populations with higher milk or meat consumption.

The food intake of participants in our study, reported from a food frequency questionnaire (FFQ) of 103 items, was similar to that reported in previous reports that used different methods to assess food intake^21,23,26^. Mean fruit intake reported from 24-hour recalls of the KNHANES 2013, a nationally representative database of non-institutionalized Koreans, was 219 g/d for adults 50–64 years old and 147 g/d for adults ≥65 years old^27^. This equals approximately 1–2 servings/day or 7–14 servings/week. (One serving of fruit is defined by the energy content, 50 kcal, which equals 150 g for watermelon and strawberries, and 100 g for most other fresh fruit and all fruit juices^17^.) The median fruit intake in our study population was similar (13 servings/week). Regarding mean vegetable consumption in adults 50–64 and ≥65 years old was 374 g/d and 310 g/d, respectively, when assessed by a 24-hour recall in the KNHANES^26^, which equals approximately 31.5–49 servings/week. (One vegetable serving size is 50 kcal of vegetables^17^, which is approximately 70 g for most vegetables and ≤40 g for burdock, seaweeds, and mushrooms in the Korean diet.) Similarly, the median vegetable intake in our study population was 45.3 servings/week. Milk intake in our population was slightly lower than those of the Health Examinees participants. We were unable to find reports on unprocessed meat consumption in different Korean cohorts. Still, there is little evidence that food consumption of our study population could have deviated from that of normal Korean older adults.

Our study has some limitations. First, the 10-year follow-up in adults ≥40 years old may not be adequate to assess the long-term effect of exposure. The Korea Centers for Disease Control and Prevention (KCDC) is continuing to collect follow-up data from these adults, which may be analyzed in the future. In contrast, because the onset of type 2 DM commonly occurs around 40 years of age, the interaction between food intake and genetics needs to be examined earlier to prevent or alleviate the risk of DM. By excluding participants with DM at baseline, we may have underestimated the association and interaction by eliminating a large population that may potentially prevent or delay the onset of DM by altering food intake according to one’s genetic variation. However, due to the lack of younger participants at baseline in the Korean Genome and Epidemiology Study (KoGES) database, we were unable to assess the genotype association with DM incidence modified by food/nutrient intake in younger populations (<40 years). Second, though FFQs are the most commonly used measurement for average food intake, the accuracy of FFQs may be limited due to its reliance on memory. However, in our prospective cohort study, dietary intakes were assessed prior to DM diagnosis and thus the resulting measurement error is most likely independent of outcome (i.e., non-differential) and may have only attenuated the association. Third, the function of ANGPTL3 has been reported to be closely associated with ANGPTL8, which is known to promote the ability of ANGPTL3 to bind and inhibit LPL *in vitro*^28^. However, we were unable to investigate the interaction between *ANGPTL3* rs11207997 and *ANGPTL8* due to limited data. Despite these limitations, this study evaluated associations with clinical endpoint (vs. intermediate biochemical endpoints) and disease incidence (vs. prevalence) using a prospective cohort design. The prospective nature of this 10-year follow-up study reduces disease-related recall bias and further supports a possible causal effect.

In summary, we found that among older Koreans, T allele carriers of rs11207997 in *ANGPTL3* had a lower risk of DM, possibly through a lifelong set point of circulating TG. In addition, among those who habitually consume fruits, vegetables, or sodium at or near the recommended intakes, adults with the T allele have lower HRs for DM than CC carriers. The association of the genetic variants of rs11207997 with the risk of DM may be masked when food or sodium intakes are far from the recommended intakes.

## Methods

### Study population

**Figure 2:**
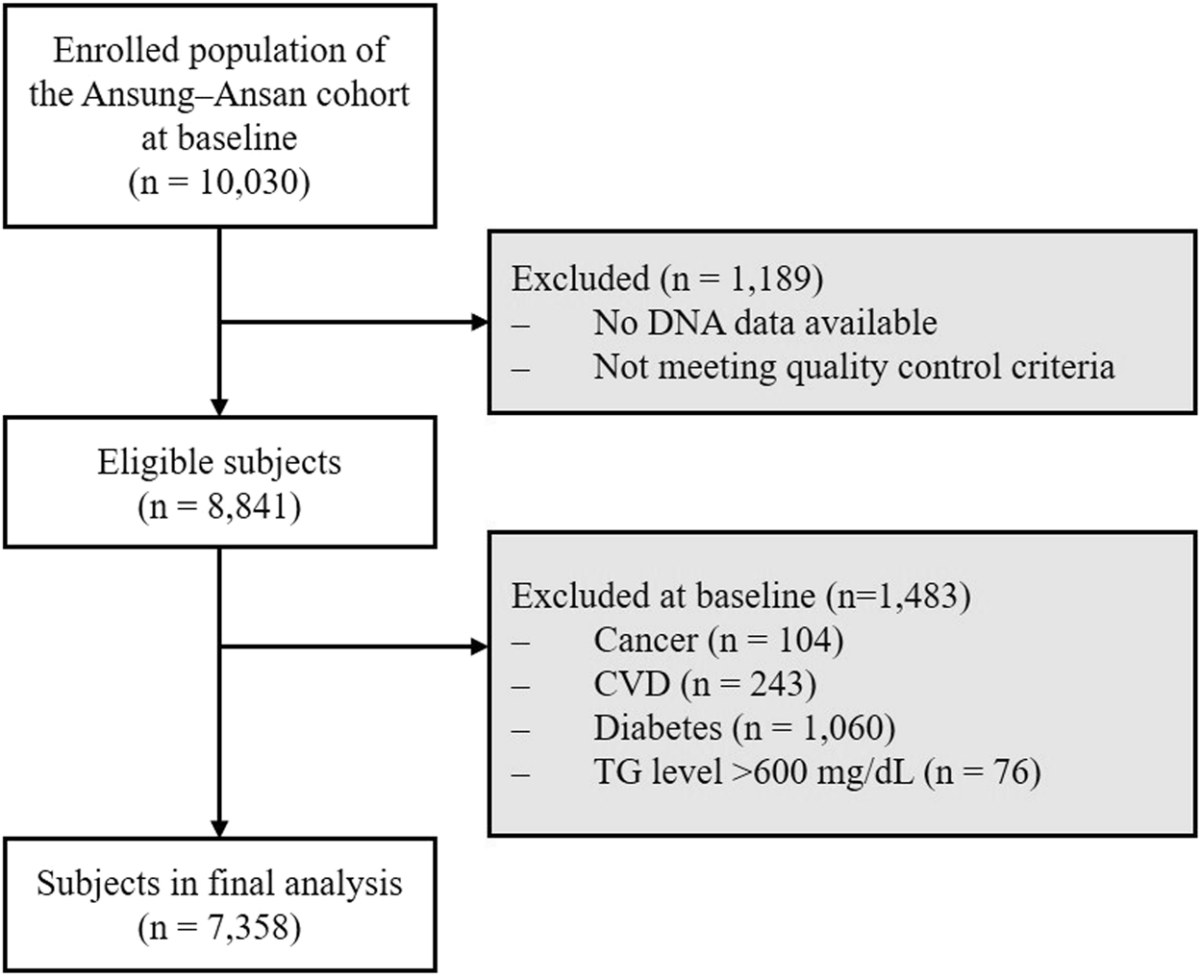
Flow chart of study subject selection. CVD: cardiovascular disease, TG: triglyceride.

Data for this study were obtained from the Ansan–Ansung cohort, which was part of the KoGES. Detailed information about the KoGES has been published in a previous study^29^. Briefly, the baseline survey of the Ansan–Ansung cohort was performed in 2001–2002 with a total of 10,030 participants aged 40–69 years, and follow-up survey data were collected biennially. Person-years for each participant was estimated from the baseline examination until the date of the first DM diagnosis at the hospital, the date of the last contact, or the date of the last follow-up visit (November 2012), whichever happened first. Each investigation data record included demographic information, lifestyle characteristics, medical anthropometric and biochemical measurements, history, and disease incidence data. Our study focused on 8,841 participants with finalized DNA genotyping and quality control data (Fig. 2). Among these 8,841 participants, individuals with cancer (*n* = 104), CVD (*n* = 243), DM (*n* = 1,060), and high TG levels (>600 mg/dL) (*n* = 76) at baseline were excluded from the study, leaving a total of 7,358 participants (3,427 men and 3,931 women) whose data were included in the final analysis. Informed consent was provided by all participants. The study protocol was approved by the Institutional Review Board of the KCDC (KBP-2016-062) and the Institutional Review Board at Korea University (KU-IRB-16-EX-272-A-1) and all methods were carried out in accordance with the approved protocol and the relevant guidelines. This study is not a clinical trial.

### General characteristics

At baseline and each follow-up examination, study participants were asked to complete survey questionnaires regarding demographic and behavioral data, including age, sex, location, total energy intake, BMI, physical activity, income level, education level, cigarette smoking, and alcohol use. Total daily energy intake (kcal) was estimated by a semi-quantitative FFQ, which was validated by the KoGES^30^. BMI (kg/m^2^) was calculated as the weight in kilograms divided by the square of the body height in meters. Height was measured to the nearest 0.1 cm. Weight, with participants in light clothing, was measured to the nearest 0.1 kg. Total metabolic equivalent (MET; hours/day) was used for measuring the energy cost of physical activities, calculated by summing the MET values of each physical activity type (2.4 for light, 5.0 for moderate, and 7.5 for intense activities)^31,32^. Monthly household income level was categorized into lowest (<1 million Korean won), lower-middle (1–2 million), upper-middle (2–4 million), and highest (>4 million). Education level was categorized into elementary school, middle school, high school, and university, considering the highest level of education completed by the participants. Smoking and drinking status were categorized into current smokers/drinkers (defined as those who answered on the questionnaire as smoking cigarettes or drinking alcoholic beverages currently) and non-smokers/drinkers.

### Dietary information

Dietary information was investigated using a semi-quantitative FFQ at the baseline survey (2001–2002) and at the second follow-up (2005–2006), which comprised questions on 103 food items. The questionnaire responses for frequency of food consumption and serving size of each food item were collected, and weighted food frequencies for each parameter were derived. To identify the effect modifier regarding associations between *ANGTPL*3 polymorphisms and DM, we selected dietary intake as the average of two surveys for fruit, vegetables, whole grains, unprocessed red meat, sodium, and milk. Fruit intake was the sum of the weekly consumption of persimmons, mandarin oranges, melons, bananas, pears, apples, oranges, watermelons, peaches or plums, strawberries, and grapes. Vegetable intake was the sum of the weekly consumption of tomatoes, green peppers, pepper leaves, spinach, lettuce, perilla leaves, chives or water parsley, other green vegetables, radishes, balloon flowers or deodeok (*Codonopsis lanceolata*), onions, cabbages, cucumbers, bean sprouts, carrots, pumpkins, bracken or sweet potato vines, kimchi (salted and fermented vegetables: cabbage kimchi, diced radish kimchi, radish kimchi, water kimchi, or other kimchi), pickled vegetables, and mushrooms. Whole grain intake was the sum of the weekly consumption of barley rice, multi-grain rice, flour made of mixed grains, and noodles made of buckwheat. Unprocessed meat intake was calculated as the sum of the weekly consumption of pork (roast pork ribs and sirloin, pork belly, and steamed pork), beef (roast beef, beef soup, beef stew, and beef ribs), unprocessed poultry (chicken legs, chicken wings, and other chicken meat), canine meat, and organ meat. Daily sodium intake was calculated based on the Standard Tables of Food Composition of Korea^22^.

### Anthropometric and biochemical measurements

Study data included biochemical measurements, for which detailed procedures and analysis methods have been described in a previous study^33^. BP was measured in both arms using a mercury sphygmomanometer. Two measurements were made with the arm above the heart level in a sitting position after at least 5 min of rest, and the average of the two was used for systolic and diastolic BP (SBP and DBP; mmHg). Blood samples for biochemical analysis were collected after fasting for at least 8 h. TG (mg/dL), TC (mg/dL), HDLC (mg/dL), FBG (mg/dL) and the oral glucose tolerance test (OGTT)−120 levels were measured using an automatic analyzer (ADVIA 1650 and 1680; Siemens, Tarrytown, NY, USA). In this study, we used the Friedewald equation for calculating LDLC (mg/dL): LDLC = TC − TG/5 − HDLC in participants with TG <400 mg/dL^34^. HbA1c was determined from whole blood samples. Blood glucose and insulin concentrations were measured at 60 and 120 min during the OGTT. The following equation was used to calculate HOMA-IR: HOMA-IR = [fasting glucose (mmol/L) × fasting insulin (μIU/mL)]/22.5^35^.

### Genotyping information

Detailed information regarding DNA preparation, genotyping, and quality control in the KoGES has been reported elsewhere^33^. Briefly, genomic DNA was isolated from the peripheral blood of all participants and genotyped on the Affymetrix Genome-Wide Human SNP Array 5.0 (Affymetrix, Inc., Santa Clara, CA, USA). Bayesian robust linear modeling using the Mahalanobis distance genotyping algorithm was used for genotyping accuracy. A total of 352,228 SNPs were available after quality control with a high missing gene call rate (>5%), low minor allele frequency (<0.01), considerable deviation from the Hardy–Weinberg equilibrium (HWE; P < 1 × 10^−6^), and sex mismatch. After rejecting an additional 48,003 SNPs outside the HWE (P < 1 × 10^−5^), a subset of 304,225 SNPs was processed by the EIGENSTRAT software package^36^. To test its association with DM, we selected a gene variant of *ANGPTL3* (rs11207997).

### Definition of DM

DM was defined by self-reported information from a biennial questionnaire implemented by trained technicians. Participants were defined to have DM if (i) they had been diagnosed with DM by a physician, (ii) their FBG level was ≥126 mg/dL, (iii) their OGTT−120 glucose level was ≥200 mg/dL, or (iv) they were taking diabetes medication.

### RNA extraction and semi-quantitative reverse transcription

Total RNA was isolated from LCLs using the Ribospin^TM^ Kit (Gene All, Korea), according to the manufacturer’s protocol. To demonstrate the relationship between the mRNA expression of *ANGPTL3* genetic variants and cardiometabolic parameters, LCLs of 62 healthy subjects (24 men and 38 women) were collected from the Ansan–Ansung cohort of the KoGES (Institutional Review Board no. KBP-2016-062, KU-IRB-16-EX-272-A-1). We obtained cDNA from 1 μg of RNA using oligo-dT and superscript II reverse transcriptase (Invitrogen, USA). One microgram of cDNA was subjected to quantitative real-time polymerase chain reaction (PCR) amplification using the SYBR Green PCR kit (Qiagen, USA). The primer sequences were as follows: ANGPTL3 (sense, 5′-TCT CCA GAG CCA AAA TCA AGA T-3′; antisense, 5′-TTT CAC TGG TTT GCA GCG AT-3′) and glyceraldehyde 3-phosphate dehydrogenase (GAPDH; sense, 5′-TCC ACC ACC CTG TTG CTG TA-3′; antisense, 5′-ACC ACA GTC CAT GCC ATC AC-3′). The PCR program was conducted under the following conditions: 15 min at 95 °C, followed by 40 thermal cycles at 94 °C for 30 s, 60 °C for 20 s, and 72 °C for 30 s. Semi-quantitative reverse transcription-PCR and quantification of gene expression were performed using QuantStudio™ 6 Flex Real-Time PCR System (Applied Bio systems, Foster City, CA). Data were analyzed using the comparative cycle threshold method, and the GAPDH expression value was used as a reference for normalization.

### Statistical analyses

Data are represented as mean ± standard error for continuous variables and percentages and the number of counts for categorical variables. Main general characteristics and biochemical parameters were compared according to DM using Student’s t-test and chi-square tests for continuous and categorical variables. Data for the statistical analyses of TG were used after log-transformation because TG was not normally distributed in this population. Differences of clinical parameters according to *ANGPTL3* genotype were obtained using analysis of variation and the generalized linear regression model with Bonferroni correction after adjusting for age, sex, location, total energy intake, BMI, physical activity, education level, and drinking status. The relationships between the *ANGPTL3* mRNA level and TG, TC, HDLC, LDLC, SBP, and DBP were tested by partial correlation analyses with adjustments for potential covariates. Results are presented as estimated correlation co-efficient (r) with *p*-values for continuous variables. The Cox proportional hazards model was used to estimate the genetic effect of *ANGPTL3* on the risk of developing DM. The Cox regression model was adjusted for age, sex, location, total energy intake, BMI, physical activity, education level, smoking and drinking status. Results are shown as HRs with 95% CI and corresponding *p*-values. Because circulating levels of TG at baseline could be an intermediate variable through which rs11207997 is associated with DM, we adjusted for them only in the secondary analyses. We also performed stratification analyses in the Cox hazard model by dietary risk factors in evaluating the associations of rs11207997 (*ANGPTL3*) polymorphism with DM incidence. All statistical analyses were performed using Stata SE 13.0 (Stata Corp., Carolina, USA).
